# Fabrication of Caseinate Stabilized Thymol Nanosuspensions via the pH-Driven Method: Enhancement in Water Solubility of Thymol

**DOI:** 10.3390/foods10051074

**Published:** 2021-05-12

**Authors:** Wei Zhou, Yun Zhang, Ruyi Li, Shengfeng Peng, Roger Ruan, Jihua Li, Wei Liu

**Affiliations:** 1 State Key Laboratory of Food Science and Technology, Nanchang University, Nanchang 330047, China; weizhou111@foxmail.com (W.Z.); zhangyun@email.ncu.edu.cn (Y.Z.); ruanx001@umn.edu (R.R.); liuwei@ncu.edu.cn (W.L.); 2 Key Laboratory of Tropical Crop Products Processing of Ministry of Agriculture and Rural Affairs, Agricultural Products Processing Research Institute, Chinese Academy of Tropical Agricultural Sciences, Zhanjiang 524001, China; mwlry19901016@163.com (R.L.); foodpaper@126.com (J.L.); 3 School of Life Sciences, Nanchang University, Nanchang 330031, China

**Keywords:** thymol, nanosuspensions, caseinate, pH-driven method, water solubility

## Abstract

Thymol has been applied as a spice and antibacterial agent in commercial products. However, the utilization of thymol in the food and pharmaceutical field has recently been limited by its poor water solubility and stability. In this work, a caseinate-stabilized thymol nanosuspension was fabricated by pH-driven methods to overcome those limitations. Firstly, the chemical stability of thymol at different pH value conditions was investigated. The physiochemical properties of thymol nanosuspensions were then characterized, such as average particle size, zeta potential, encapsulation efficiency, and loading capacity. Meanwhile, the X-ray diffraction results showed that thymol was present as an amorphous state in the nanosuspensions. The thermal stability of thymol was slightly enhanced by encapsulation through this process, and the thymol nanosuspensions were stable during the long-term storage, and the average particle size of nanosuspensions showed that there was no aggregation of nanosuspensions during storage and high temperature. Finally, the antimicrobial activity of thymol nanosuspensions was evaluated by investigating the minimum inhibitory concentration (MIC) and minimum bactericidal concentration (MBC) against *Salmomella enterca*, *Staphlococcus aureus*, *Escherichia coli*, and *Listeria monocytogenes*. These results could provide useful information and implications for promoting the application of thymol in food, cosmetic, and pharmaceutical commercial products.

## 1. Introduction

Thymol (2-isopropyl-5-methylphenol), a monoterpene phenol, is the principal active component of essential oil obtained from thyme, which has been applied as traditional herbs in the cure of disorders affecting the digestive, cardiovascular, and nervous systems [1]. Due to the numerous functions of thymol, such as antioxidant, anti-inflammatory, antimicrobial, and anticarcinogenesis functions, the research on its potential to be utilized as a food preservative and nutraceutical is increasing dramatically [2]. For example, Pan et al. stated that thymol could be utilized in food matrices and depolarize the microbial cytoplasmic membrane to extend the shelf life of productions [3]. Moreover, thymol is already widely applied as a natural feed additive for livestock, which could improve the metabolism and absorption of the nutrients in the animal gut via enhancing digestive enzymes and modulating intestinal microbiota activity to increase the growth indices (including body weight, feed efficiency, and daily growth rate) of them [4,5,6]. Thymol has been included in the list of ‘Generally Recognized As Safe’ for application as food additives by the FDA, and it was registered by the European Commission for utilization in the food field due to the lack of intake risk [7,8]. Meanwhile, a comprehensive review has concluded and shown the safety and anticancer activity of thymol [9]. However, its commercial application is limited due to its hydrophobic nature and low water solubility [8,10]. Several types of delivery and encapsulation systems have been investigated and evaluated to enhance the water solubility of thymol; for example, Nasrabadi et al. [11] improved the water solubility and bioaccessibility of thymol via encapsulating it into Pickering emulsions; Cakir et al. [12] fabricated thymol loaded chitosan nanoparticles via ionic gelation method to overcome the limitation of thymol; and nanoliposome was applied by Heckler et al. [13] to encapsulate free thymol as antimicrobials in food products. Normally, thymol as a lipophilic element has been encapsulated in delivery systems via dissolving thymol into organic liquid firstly, which is criticized as leading to high costs and potential damage to the consumer’s health. Thus, it is significant to find effective and environmentally friendly methods for handling the key application limitation and challenge of thymol encapsulation.

Recently, an easily scalable and environmentally friendly approach, termed the pH-driven method, was developed [14,15]. It has been utilized to incorporate lipophilic phenols (mainly curcumin) into a variety of delivery systems such as liposomes [16], nanoparticles [17,18], emulsions [19], and oil body [20]. The mechanism of this method is controlling the water solubility of lipophilic phenols via adjusting the pH values. At lower pH values, the lipophilic phenols showed poor water solubility. When the pH value was higher than the pKa of the hydroxyl group of phenols, the deprotonation of the hydroxyl group leads to an increase in the charge and water solubility of phenols. Then, the deprotonated/water-soluble phenols become protonated/water insoluble once the pH value is lower than the pKa. These protonated lipophilic phenols molecules show different behaviors in different systems: (1) In pure water systems, these molecules recrystallize into crystal nucleus, which grows into phenol crystal and precipitates [21]; (2) in the presence of biosurfactant or biopolymers, these amphiphilic molecules are absorbed onto the surface of the crystal nucleus and prevent its growth, and biosurfactant/biopolymer coated phenol nanoparticles are formed [15,17,22]; (3) in the presence of delivery systems with hydrophobic domains (such as liposomes, emulsions, and zein nanoparticles), the protonated lipophilic phenol molecules penetrate into hydrophobic domains of delivery systems [23]. Among these phenol loaded delivery systems fabricated by the pH-driven method, biosurfactant/biopolymer-stabilized phenol nanosuspensions showed great potential due to their easier procedure and higher loading capacity. While the nanosuspensions showed great advantages, they were mainly applied to encapsulate curcumin. Whether they could be applied to encapsulate other lipophilic phenols is still unknown. The influence of phenol properties on the formation and structure of phenol nanosuspensions needs to be explored.

Caseinate as the main source protein obtained from milk has been widely applied in food industries due to its desirable amphiphilic properties and abundant essential amino acids [24]. In previous research, caseinate has been employed concerning hydrophobic nutrients, such as curcumin, beta-carotene [25], and quercetin, which could improve their stability and water solubility. Compared with some other synthetic compounds, for instance, inorganic materials and small molecule surfactants, caseinate as a kind of natural material is more easily accepted by consumers and food factories. Our previous research indicated that caseinate is a wonderful encapsulation material for lipophilic nutrients via the pH-driven method with the highest encapsulation efficiency and loading capacity compared with serval kinds of proteins and polysaccharides [17]. All of this information proved the potential of caseinate as shell and encapsulation compounds to construct nanoparticles with thymol. However, the relevant results and information of nanoparticle fabrication with thymol and caseinate via the pH-driven method still need to be reported.

Therefore, the main objective of this study was to fabricate thymol nanosuspensions stabilized by caseinate via the pH-driven method and to check if the pH-driven method is suitable for thymol nanosuspensions. First, the chemical stability of thymol in different pH value conditions was evaluated. Then, the caseinate-stabilized nanosuspensions were prepared through the pH-driven method, and the stability, encapsulation efficiency, and particle properties were determined. Finally, the antibacterial properties of encapsulated thymol were also investigated. These results will provide important information for the encapsulation of thymol via the pH-driven method, which is useful for application in the fabrication and design of food cosmetics and pharmaceutical products.

## 2. Materials and Methods

### 2.1. Materials

Thymol and caseinate powder were purchased from Aladdin Biochemical Technology Co., Ltd. (Shanghai, China). Bacterial strains (CICC 22956, CICC 21600, CICC 10003, CICC 21635) were obtained from China General Microbiological Culture Collection Center (Beijing, China). All other chemicals were of analytical grade.

### 2.2. Preparation of Nanosuspensions

Thymol nanosuspensions were prepared by a pH-driven method according to our previous study with some modification [15,22]. Thymol powder was weighed and dissolved in 0.1 M NaOH to obtain a thymol alkaline solution (10 mg/mL). Caseinate stock solutions (50 mg/mL) were prepared by dissolving caseinate in PBS (5 mM, pH 6.5) for 4 h and the solution was centrifuged at 8000 rpm for 30 min to remove the undissolved impurities. The solution was then diluted with PBS to different concentrations and stored at 4 °C overnight before use. Thymol alkaline solutions were then added into the caseinate solution (1:1, *v/v*) with constant stirring using a magnetic stir plate. Nanosuspensions with a thymol concentration of 5 mg/mL and different caseinate concentrations (2.5, 5, 10, 15, 20, and 25 mg/mL) were prepared by adjusting the pH value to 6.5 using 6 M HCl solutions.

### 2.3. The Physical Properties of Nanosuspensions

The average particle size and surface potential of nanosuspensions were determined by Zetasizer Nano ZSP (Malvern Instruments Ltd., Worcestershire, UK) at room temperature. Samples were diluted tenfold with PBS, and each sample was tested three times in parallel.

The microstructure of nanosuspensions was observed using Atomic Force Microscopy (AFM, C300, Nanosurf, Liestal, Switzerland). The nanosuspension solutions were diluted 1000-fold with distilled water and one drop of samples was placed on a freshly cleaved mica substrate. The images of the sample were obtained using the AFM operated with a silicon cantilever force constant of 0.58 N m^−1^ in tapping mode. 

The morphology of the nanosuspensions was further confirmed using transmission electron microscopy (TEM). Briefly, nanosuspensions were placed onto a copper mesh grid for 4 min. The sample was then stained with 1% uranyl acetate solution for 1 min and then washed with double distilled water. The sample-loaded grid was then air dried at room temperature and imaged using a TEM (JEM-2000FX, JEOL, Ltd., Tokyo, Japan) operating at a voltage of 200 kV.

The crystalline properties of nanosuspensions were investigated using X-ray diffraction. The blank casein nanoparticles (without thymol) fabricated using the pH-driven method and thymol powder were used as control groups. The emission slit was 1°, the accepted slit was 0.1 mm, and the scanning speed was 2°/min. 

The loading capacity (LC) and encapsulation efficiency (EE) were calculated according to the previous method [2]. Samples were extracted with n-hexane (volume ratio 1:9) and the absorbance was then measured at 263 nm using a UV–visible spectrophotometer. The thymol concentration was then calculated using a standard curve prepared. Then, the insoluble part of the thymol was removed by centrifugation (6000 rpm, 10 min), and the thymol in the supernatant fluid was extracted by n-hexane to measure the amount of dissolving thymol in the samples. The free thymol was separated and measured via an ultrafiltration method (3 kDa). The EE and LC of samples were calculated with the following formulas:

EE (%) = (W_s_ − W_f_)/W_t_ * 100 (1)

LC (%) = (W_s_ − W_f_)/M * 100 (2)

where W_t_ represents the total thymol content, W_s_ represents the dissolved thymol content, and W_f_ represents the free thymol content. M is the total mass of the loaded nanosuspensions: Thymol and caseinate.

### 2.4. The Stability of Thymol Nanosuspensions

Thermal stability: Nanosuspensions were treated at 80 °C for 1 h and sampled every 10 min to measure the properties (retention rate, average diameter, and visual pictures) according to previous methods of nanosuspension after heating.

Storage stability: Samples were stored at room temperature (25 °C) for one month, and their properties (retention rate and average diameter) were investigated every 7 days.

### 2.5. The Antibacterial Properties of Nanosuspensions

LB liquid medium: 10 g tryptone, 5 g yeast extract, and 5 g sodium chloride were weighed and dissolved in 1000 mL distilled water. The medium was autoclaved at 121 °C for 20 min. The medium was placed at 4 °C for use after cooling.

LB solid medium: 10 g tryptone, 5 g yeast extract, 5 g sodium chloride, and 2 g agar were weighed in 1000 mL distilled water, autoclaved at 121 °C for 20 min, and then poured into 90 mm Petri dishes with about 12–15 mL in each dish. After cooling and solidification, they were placed upside down at 4 °C.

Bacterial resuscitation and rejuvenation: First, 50 µL of glycerin bacteria were vaccinated in 5 mL of LB liquid medium and cultured in a biochemical incubator at 37 °C overnight to turbidity, and then the strains were inoculated from the LB liquid medium to the LB solid medium using inoculation loops to an incubator at 37 °C constant temperature for 10–12 h. Then, the appropriate single colony was inoculated to a liquid medium and cultured to a later growth stage. The bacterial strains were transferred to a solid medium again, sealed by film, and stored at 4 °C with the appropriate single colony observed.

Bacterial culture: The plate was taken out of a refrigerator at 4 °C; a single colony was inoculated in a 5 mL LB liquid medium and cultured in a constant temperature vibration incubator at 37 °C and 220 rpm. For the minimum inhibitory concentration and maximum bactericidal concentration, the bacterial solution was diluted to 10^6^ CFU/mL.

MIC measurement: The 96-well microplates method was adopted according to the previous method [13]. The test bactericidal strains were CICC 22956, CICC 21600, CICC 10003, and CICC 21635. First, 100 µL of liquid LB medium was added to each well. Next, 100 µL samples were placed into the first row of the wells and mixed, 100 µL of the mixture was placed into the next well, and so forth. Then, 100 µL bacterial solutions were mixed into each well and incubated (37 °C, 24 h) and put into an incubator at 37 °C for culture for 24 h. The minimum sample concentration with clear culture and no substrate precipitation was the minimum inhibitory concentration (MIC).

MBC measurement: 100 µL was obtained from the MIC well and inoculated in culture, then the cultures were placed in an incubator at 37 °C for 12 h. The sample concentration corresponding to the plate without bacterial colony was the maximum bactericidal concentration (MBC).

### 2.6. Statistical Analysis

All of the experiments were repeated at least three times, and the results were expressed as mean ± standard deviation. SPSS 18.0 software and the Student–Newman–Keuls (SNK) equation were applied to analyze the significance (*p* < 0.05).

## 3. Results and Discussion

### 3.1. Fabrication of Nanosuspensions

**Figure 1:**
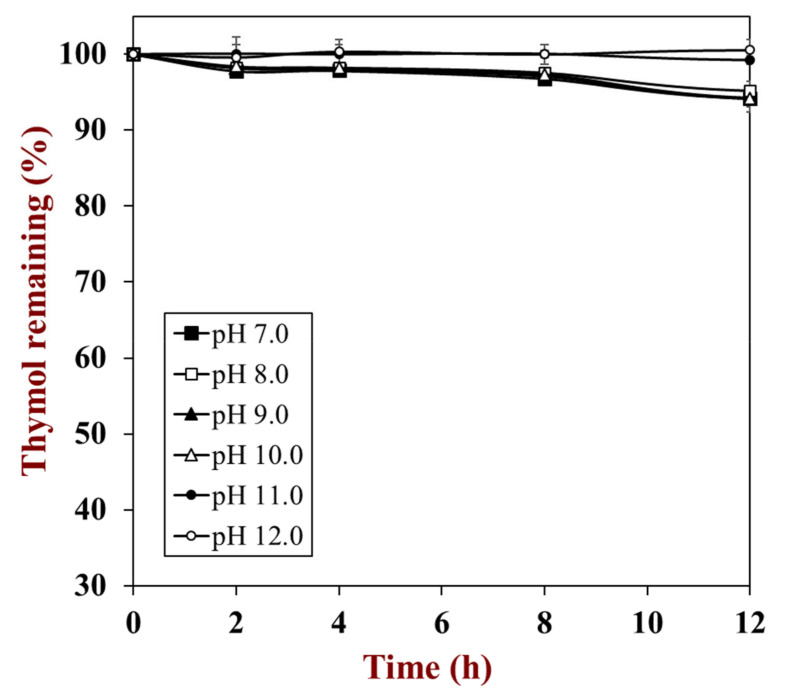
The influence of pH values on the stability of thymol.

During the fabrication of thymol nanosuspensions through the pH-driven method, thymol must be dissolved in the alkaline solutions for at least 5 min. When thymol was dissolved in alkaline solutions, the phenolic hydroxyl group that was thymol ionized and the potential charge of the thymol molecule became negative, which caused an enhancement in its hydrophilic properties and water solubility. It is well known that phenols are unstable under alkaline conditions [26,27]. Therefore, the chemical stability of thymol was firstly investigated via measuring the retention concentration at different pH conditions. As displayed in Figure 1, the retention rate of thymol at a pH range of 7.0 to 10.0 slightly decreased to 93.0% after 24 h, while thymol at pH 11.0 and 12.0 was ultra-stable without any degradation. During the pH-driven process, the thymol was dissolved in alkaline solutions (around pH 12.5), which meant the loss of thymol during the pH-driven process was negligible. 

Thymol showed relatively high chemical stability compared with other phenols. Our previous study showed that the chemical stability of phenols negatively corresponded to the number of hydroxyl groups [28]. Thymol molecules have only one hydroxyl group, which could explain why they showed extremely high stability. On the other hand, thymol showed higher stability at pH 11.0 and 12.0 compared to lower pH values (7.0 to 10.0), which was similar to curcumin [29]. The chemical stability of quercetin and resveratrol, however, negatively corresponded to the pH values (i.e., the higher the pH, the poorer the chemical stability). The difference could be assigned to the presence of the −O−CH_3_ group or the −CH−(CH_3_)_2_ group in curcumin and thymol molecules, respectively. There are no other groups in the quercetin and resveratrol except the hydroxyl group.

**Figure 2:**
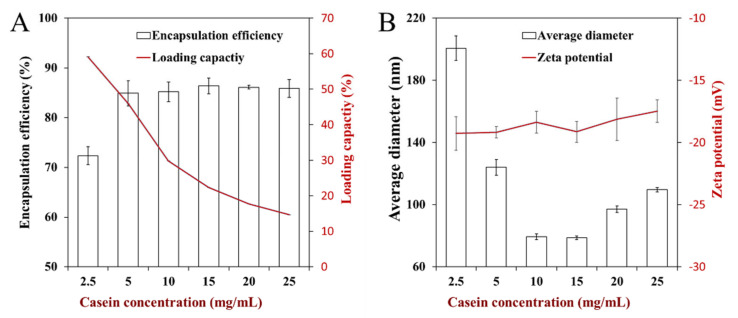
The influence of casein concentration on encapsulation efficiency and loading capacity (**A**), and average diameter and zeta potential (**B**) of thymol nanosuspensions.

Initially, the influence of caseinate concentration on the encapsulation efficiency and loading capacity was investigated (Figure 2A). The EE increased with the increase of caseinate concentration from 2.5 to 5 mg/mL and then became steady. The initial low EE of samples could be mainly due to the insufficient caseinate that could not form enough particles for the encapsulation of free thymol, and the free part of thymol was removed by centrifugation. At high and sufficient caseinate concentration, most of the thymol nanosuspensions could be completely coated by caseinate during the change of pH values, and the EE and amount of encapsulated thymol increased. Since part of thymol could be dissolved in water, even the extra protein and nanosuspensions were present with caseinate increasing; no significant increase of EE could be observed. To further investigate the encapsulation level of thymol into nanosuspensions, the influence of caseinate concentration on the LC was also evaluated (Figure 2A). The LC of thymol nanosuspensions decreased with the increase of caseinate concentration, which could again be due to the hypothesis that most of the thymol had been encapsulated. The largest LC of thymol nanosuspensions coated by caseinate was nearly 60%; this phenomenon and result proved that the nanosuspensions coated by caseinate are extremely suitable encapsulation systems for thymol via the pH-driven method. 

The influence of caseinate on the average particle size and zeta potential of nanosuspensions are shown in Figure 2B. It is obvious that the size of nanosuspensions dramatically decreased with the caseinate concentration increasing from 2.5 to 10 mg/mL. The decrease of nanosuspension size could be mainly attributed to the behavior and function of caseinate molecules, which are adsorbed at the surface of thymol particles via their hydrophobic regions to prevent the aggregation of nanosuspensions. When the concentration of caseinate increased from 10 to 25 mg/mL, the size of nanosuspensions increased gently, which could be due to an accumulation of caseinate at the nanosuspension surface. Conversely, there was no significate difference between the zeta potential of nanosuspensions fabricated with different concentration caseinate; all of the nanosuspensions presented a moderately negative charge. The charge of nanosuspensions could be mainly due to the properties of caseinate by which the pH values of water were higher than its isoelectric point, and the surface charge of nanosuspensions was dominated by caseinate, which covered and was present at the surface of nanosuspensions.

The ideal function and behavior of caseinate during the pH-driven method could be related to the emulsifying and binding ability of this kind of protein. It has been reported that amphiphilic biopolymers (such as whey protein isolate) can adsorb to the surface of the lipophilic nanoparticle, prevent contact, and aggregate between them [17]. These data and phenomena showed that the water solubility and dispersibility of thymol could be enhanced by encapsulation via the pH-driven method with caseinate, and this information could promote the development and application of thymol in food and cosmetic productions. 

### 3.2. Characterization of Nanosuspensions

A caseinate concentration of 10 mg/mL was used in the following studies since it led to relatively high EE and LC values with a small nanosuspension size distribution. As discussed above, the nanosuspensions with final formal were relatively small (79.4 nm) and negatively charged (−19.4 mV). At the same time, they had high EE (85.2%) and LC (30.0%), and the free thymol was dissolved in water instead of sediment. These results were relatively similar to caseinate-coated curcumin nanoparticles [17]. Previous researchers had also applied some delivery systems to encapsulate thymol and investigated the LC. For example, Shi et al. prepared thymol-loaded solid lipid nanoparticles with a maximum LC of 16% [30], and the LC of thymol/soy protein nano-complexation was 10.36% [2]. All of the data and experiments indicated that the thymol was suitable for the construction of nanosuspensions coated with caseinate via the pH-driven method. 

**Figure 3:**
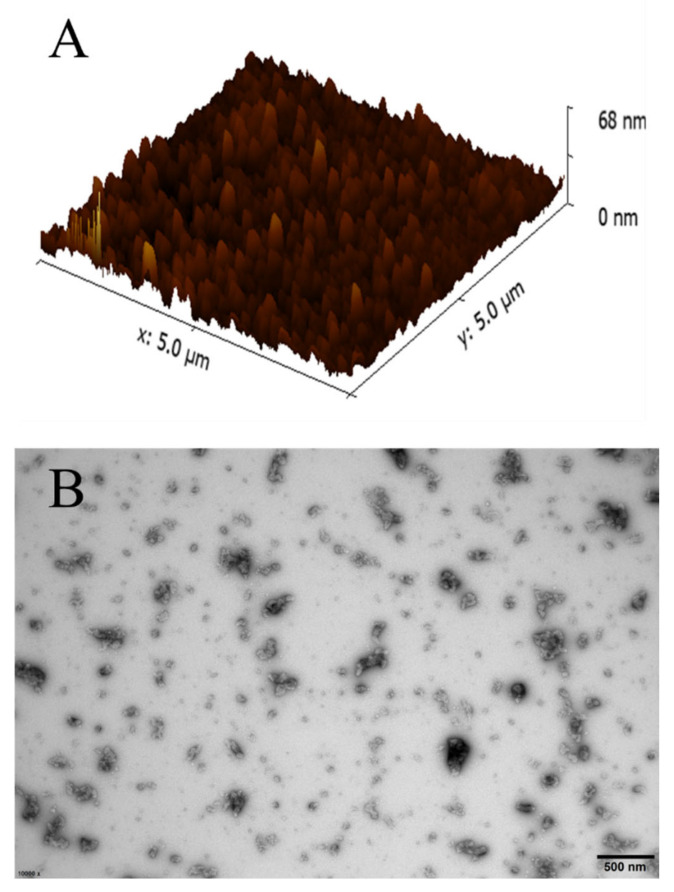
Atom force microscopy (**A**) and transmission electron microscopy (**B**) image of thymol nanosuspension.

To further prove the fabrication of nanosuspensions, their morphology was observed and detected by AFM and TEM (Figure 3). The AFM results demonstrated that the thymol nanosuspensions are spherical and uniformly distributed in the systems. As expected, the height and size of nanosuspensions were consistent with results provided by dynamic light scattering. In the TEM image, most of the particles were uniformly distributed. The sizes of nanosuspensions were in the range of 50–150 nm, which agrees with the data obtained by dynamic light scattering. 

**Figure 4:**
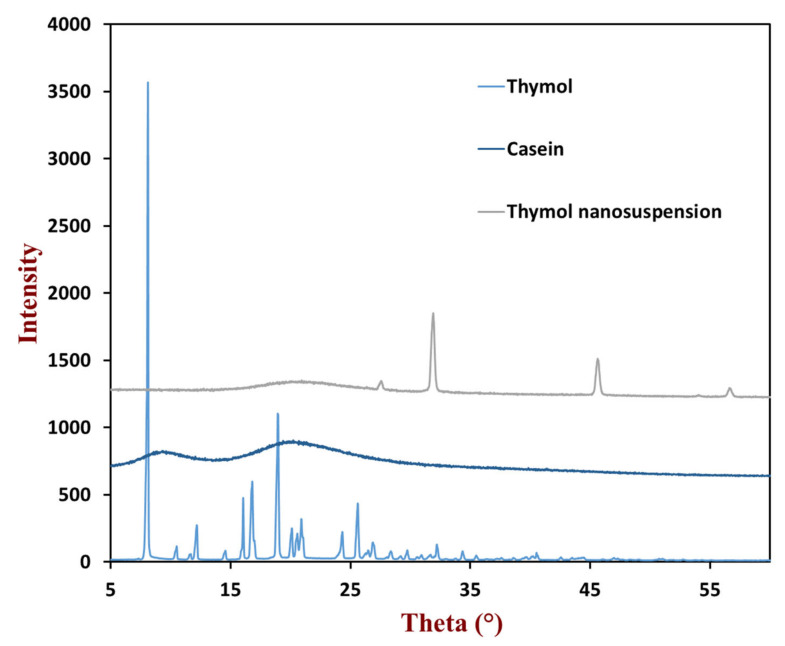
XRD spectra of thymol, casein, and powdered thymol nanosuspension.

The physical states of the thymol in caseinate-coated nanosuspensions were investigated via X-ray diffraction. The diffraction peak of thymol was obtained at 2θ values from 5° to 35° (Figure 4), which indicates the high crystal state of thymol. The relatively smooth curve of caseinate indicated that it was not present in a crystalline structure. As expected, the diffraction peak of the nanosuspensions could not be observed, indicating that all of the thymol are present in an amorphous state in the core of nanosuspensions. These phenomena and data suggest that the incorporation of thymol into the caseinate-coated nanosuspensions could prevent its crystallization, which is consistent with previous results and may be beneficial for the application in commercial products, such as food colloids.

### 3.3. Stability of Nanosuspensions

**Figure 5:**
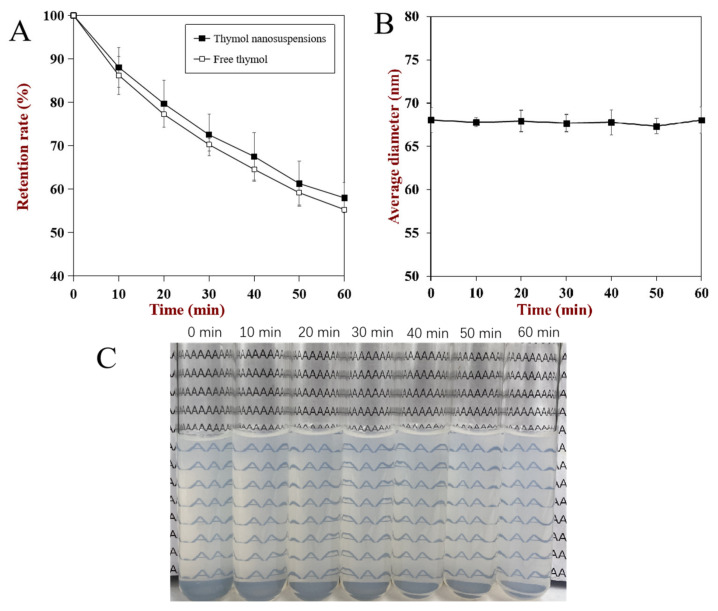
The influence of heating on the thymol retention rate (**A**), average diameter (**B**), and visual appearance (**C**) of nanosuspensions.

In food products, most nanosuspensions and food colloids are applied by incorporating them into different products and are exposed to different environmental conditions. Therefore, the thermal and storage stability of nanosuspensions were investigated in this section (Figure 5), which could provide information for the application of thymol in commercial products.

It has been reported that the volatile properties of thymol limit its application in the food industry, and the decrease of thymol mass begins from 50 °C [31]. Thus, the impact of high temperature on the stability of thymol nanosuspensions was investigated at 80 °C. The retention rate of both pure thymol and nanosuspensions decreased with time, and the retention rate of nanosuspensions was slightly higher than pure thymol. These results and phenomena indicated that the coating of caseinate at the surface of nanosuspensions could enhance its thermal stability. Similar results were also reported in nano-complexation of thymol-soy protein isolate, and Chen et al. attributed the enhancement of thermal stability to the shift of thymol evaporation temperature point from 50 to 90 °C and the inclusion complex fabrication [2,32]. Meanwhile, the influence of heat on the average size of nanosuspensions was also investigated, and it is obvious that there are no significate changes. The results of particle size indicate that there are no aggregation and dissociation of caseinate at the surface of the nanosuspensions at elevated temperature, which further proved that caseinate is suitable for the encapsulation of thymol nanosuspensions via the pH-driven method under high temperature. Meanwhile, in the visual figures (Figure 5c), all of the samples remained clear, which indicated there was no significant recrystallization and dissolution of thymol at high temperature. However, in previous research, the curcumin nanoparticles coated by caseinate via a pH-driven shift exhibited a large leakage of curcumin and a slight increase in turbidity [17]. These differences of nanoparticles are mainly due to the difference between thymol and curcumin in their molecular weight or hydrophobicity.

**Figure 6:**
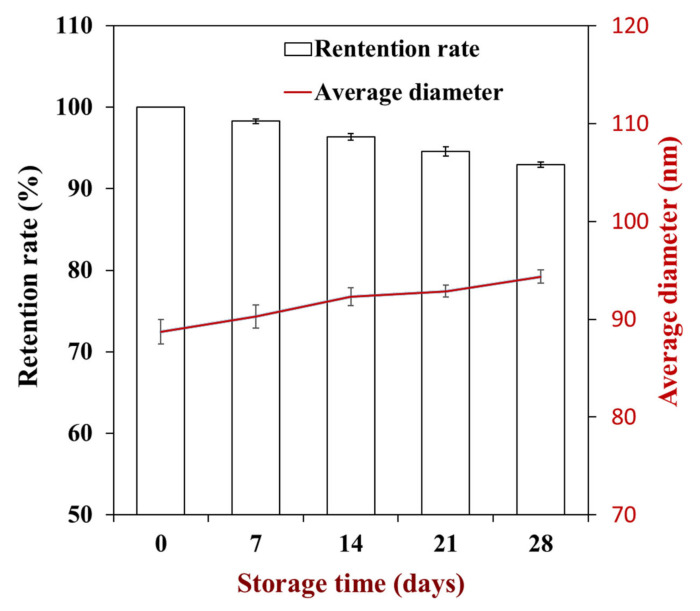
Changes of thymol retention rate and nanosuspension diameter during storage for four weeks.

To further investigate the stability of thymol nanosuspensions, their storage stability at room temperature was examined via investigating the retention rate and average diameter during 28 days of storage. While the results showed that the retention of thymol nanosuspensions decreased slowly, the value was higher than 90% during the storage period (Figure 6). Meanwhile, there were no significant changes in the average diameter of thymol nanosuspensions, which indicated that the caseinate-coated nanosuspensions via the pH-driven method were stable at long-term storage. However, it has been reported that the retention rate of curcumin nanoparticles coated by caseinate by the pH-driven method decreased dramatically during storage at room temperature [17]. This difference is the same as the thermal stability, which could be mainly due to the difference between the encapsulated lipophilic nutrients. 

### 3.4. MIC and MBC of Thymol 

As a natural antibacterial agent, thymol has been applied in many products to extend shelf life, and its antibacterial activity is better than many other plant oil components, such as carvacrol, diacetyl, and eugenol [2,13]. Moreover, it has been reported that the antibacterial activity of thymol could be mainly attributed to the thymol’s capacity to change the structure and properties of the outer and cytoplasmic membrane of bacteria [33]. Therefore, to investigate the antibacterial activity of thymol nanosuspensions, the MICs and MBCs of nanosuspensions against four common pathogenic bacteria (*Salmomella enterca*, *Staphlococcus aureus*, *Escherichia coli*, and *Listeria monocytogenes*) were investigated and calculated (Table 1).

The MICs of free thymol against four kinds of bacteria were the same as 142 mg/mL, and the values of all MBCs were the same as MICs. These results and phenomena indicated that thymol could be used as antibacterial and bacteriostatic agents for all of the four kinds of bacteria and there was no significant influence of bacteria kinds on antibacterial activity. However, all of the MICs and MBCs of thymol nanosuspensions were higher than the free thymol, which showed that there was no enhancement in antimicrobial activity of thymol by encapsulating with caseinate via pH-driven methods. These results could be due to the bonds between thymol and caseinate, which prevent the release of thymol from coated nanosuspensions. Thus, the amount of thymol in contact with bacteria decreased at the same concentration, the same antibacterial effect required a higher concentration of thymol. Similar results have also been reported by Wattanasatcha et al., that there was no difference between free thymol and thymol encapsulated in the ethylcellulose/methylcellulose sphere on its antimicrobial activity [34]. Moreover, the MIC/MBC values of thymol encapsulated into liposomes were also lower than free thymol, which has been mainly attributed to the strong binding between thymol/liposomes and the lower release of encapsulated thymol [13]. Therefore, in this work, the decrease in the amount of encapsulated thymol release could be due to the hydrophobic interaction force between thymol and caseinate, which influenced the antibacterial properties of thymol.

## 4. Conclusions

In this study, caseinate-stabilized thymol nanosuspensions were successfully fabricated via the pH-driven method. Thymol were extremely stable at pH 7.0–12.0 even after incubation for 24 h, which means the loss of thymol during the pH-driven process is negligible. The physchemical properties of thymol nanosuspensions are highly dependent on the caseinate concentration. Caseinate could stabilize thymol nanosuspensions even at a relatively low caseinate concentration, and the loading capacity can be as high as 45.9%. AFM and TEM results showed that the nanosuspensions were uniformly distributed. The thymol nanosuspensions remained stable under high temperature and long-time storage at room temperature. The antimicrobial activity of thymol nanosuspensions was lower than free thymol, which could be due to the bonding between caseinate with thymol and thus retard its release and antimicrobial effects. This work and these results may provide useful information for the design of functional food, cosmetics, and pharmaceutical productions using the pH-driven method.

## Figures and Tables

**Table 1:** Minimum inhibitory concentrations (MIC) and minimum bactericidal concentrations (MBC) of nanosuspensions and free thymol for inhibition and inactivation of *L. monocytogenes*, *S. aureusd*, *S. typhimurium*, and *E. coli*.

		Thymol Solutions	Nanosuspensions
*L. monocytogenes*	MIC (µg/mL)	142	312
	MBC (µg/mL)	142	312
*S. aureusd*	MIC (µg/mL)	142	156
	MBC (µg/mL)	142	156
*S. typhimurium*	MIC (µg/mL)	142	156
	MBC (µg/mL)	142	156
*E. coli*	MIC (µg/mL)	142	312
	MBC (µg/mL)	142	312
